# Supplementary material for: “I don’t feel safe sitting in my own yard”: Chicago resident experiences with urban rats during a COVID-19 stay-at-home order
Source: BMC Public Health. 2021 May 29;21:1008. doi: 10.1186/s12889-021-11095-y (PMC8163585; doi:10.1186/s12889-021-11095-y)
Supplement: Supplementary file 2 — Additional file 2: Supplemental tables containing model outputs (Tables S1, S2, S3, S4) and a figure showing the relationship between the change in rat sightings and sightings of rat feces in the home (Figure S1). [file 12889_2021_11095_MOESM2_ESM.pdf]

Supplemental material for manuscript "'I don't feel safe sitting in my own yard': Chicago resident experiences with urban rats during a COVID-19 stay-at-home order"

Authors: Maureen H. Murray[1,2], Kaylee A. Byers[3,4], Jacqueline Buckley[1,2], Seth B. Magle[1], Dorothy Maffei[5], Preeya Waite[6], and Danielle German[7]

Affiliations:

[1]Urban Wildlife Institute, Dept. of Conservation and Science, Lincoln Park Zoo, Chicago, IL, USA

[2]Davee Center for Epidemiology, Dept. of Conservation and Science, Lincoln Park Zoo, Chicago, IL, USA

[3]Department of Interdisciplinary Studies, University of British Columbia, Vancouver, British Columbia, Canada

[4]Canadian Wildlife Health Cooperative, Animal Health Centre, Abbotsford, BC, Canada

[5]School of Public Health, University of Illinois at Chicago, Chicago, Illinois, USA

[6]Center for Community Health Equity, DePaul University, Chicago, Illinois, USA

[7]Johns Hopkins Bloomberg School of Public Health, Johns Hopkins University, Baltimore, MD, USA

Corresponding author:

Name: Maureen H. Murray

Email: maureenmurray@lpzoo.org

Address: 2001 N Clark St., Chicago IL, 60614

Table S1: Demographic characteristics of survey respondents in Chicago, Illinois, USA during the spring 2020 stay-at-home order.

Characteristic	Survey respondents (n = 835)	Chicago 2010 census
**Age class**		
18-24	3.47%	11.20%
25-34	14.61%	19.10%
35-44	18.80%	14.00%
45-54	16.88%	12.60%
55-64	14.13%	9.80%
65+	12.69%	10.30%
NA	19.40%	-
**Gender**		
Female	54.25%	51.57%
Male	24.55%	48.53%
Non-Binary	0.60%	NA
Prefer not to say	1.08%	NA

NA	19.52%	-
**Property Ownership**		
Own	66.22%	44.91%
Rent	22.99%	55.01%
NA	10.79%	

Table S2: Ordinal regression output for variables associated with a change in rat sightings in or around the respondent's home during the quarantine period. The change in rat complaints refers to the change in 311 calls in the respondents' census tract between January and May 2020.

Variable	**β**	Std. Error	t value	p value
Restaurants	0.08	0.09	0.83	0.40
Gender (Male)	-0.01	0.18	-0.04	0.97
Age (Linear)	0.16	0.37	0.43	0.67
Age (Quadratic)	-0.45	0.31	-1.44	0.15
Age (Cubic)	-0.42	0.25	-1.70	0.09
Age (^4)	-0.02	0.20	-0.11	0.91
Age (^5)	-0.16	0.18	-0.87	0.38
Time outside (Linear)	0.49	0.23	2.18	0.03
Time outside (Quadratic)	0.11	0.19	0.59	0.56
Time outside (Cubic)	-0.01	0.15	-0.04	0.97
Renter	0.01	0.25	0.05	0.96
Housing (large multi-unit)	-0.77	0.26	-2.96	3.08 x 10-3

Housing (small multi-unit)	0.61	0.25	2.55	0.01
Median household income	0.08	0.13	0.67	0.40
Change in rat complaints	0.07	0.15	0.43	0.67

Table S3: Logistic regression output for variables hypothesized to be associated with the likelihood of a survey respondent calling 311 during quarantine.

Variable	**β**	Std. Error	z value	p value
Intercept	-1.36	0.33	-4.11	3.90 x 10-5
Change in rat sightings at home (Linear)	1.67	0.45	3.72	2.03 x 10-4
Change in rat sightings at home (Quadratic)	-0.80	0.39	-2.03	0.04
Change in rat sightings at home (Cubic)	0.31	0.32	0.98	0.33
Change in concern about rats (Linear)	2.01	0.42	4.75	2.00 x 10-6
Change in concern about rats (Quadratic)	0.04	0.37	0.12	0.91
Change in concern about rats (Cubic)	-0.06	0.32	-0.18	0.86
Change in concern about rats (^4)	-0.16	0.27	-0.59	0.55
Information about rats (Linear)	0.66	0.41	1.60	0.11

Information about rats (Quadratic)	1.27	0.37	3.45	5.63 x 10-4
Information about rats (Cubic)	0.21	0.29	0.73	0.47
Information about rats (^4)	0.34	0.24	1.41	0.16
Gender (Male)	-0.30	0.26	-1.14	0.26
Children (Yes)	0.30	0.30	1.02	0.31
Age (Linear)	0.28	0.73	0.39	0.70
Age (Quadratic)	-0.11	0.65	-0.17	0.86
Age (Cubic)	0.03	0.48	0.07	0.95
Age (^4)	-0.12	0.35	-0.34	0.73
Age (^5)	0.11	0.27	0.41	0.68
Renter	-1.13	0.40	-2.78	0.01
Median household income	-0.11	0.12	-0.96	0.34

Table S4: Ordinal regression output for variables hypothesized to be associated with the likelihood of a survey respondent calling a pest control professional during quarantine.

Variable	**β**	Std. Error	z value	p value
Intercept	-1.08	0.30	-3.58	3.42 x 10-4
Change in rat sightings at home (Linear)	0.81	0.36	2.26	0.02
Change in rat sightings at home (Quadratic)	-0.68	0.32	-2.13	0.03
Change in rat sightings at home (Cubic)	0.35	0.28	1.25	0.21
Change in concern about rats (Linear)	1.34	0.37	3.57	3.52 x 10-4
Change in concern about rats (Quadratic)	0.06	0.33	0.20	0.84
Change in concern about rats (Cubic)	-0.34	0.32	-1.07	0.29
Change in concern about rats (^4)	0.21	0.27	0.77	0.44

Information about rats (Linear)	0.23	0.40	0.57	0.57
Information about rats (Quadratic)	1.17	0.36	3.27	1.07 x 10-3
Information about rats (Cubic)	0.09	0.28	0.33	0.74
Information about rats (^4)	-0.01	0.24	-0.03	0.98
Gender (Male)	-0.08	0.25	-0.30	0.77
Children (Yes)	0.08	0.29	0.29	0.77
Age (Linear)	-0.12	0.74	-0.16	0.87
Age (Quadratic)	0.17	0.65	0.26	0.80
Age (Cubic)	-0.09	0.48	-0.18	0.86
Age (^4)	0.04	0.36	0.11	0.91
Age (^5)	0.27	0.27	1.01	0.31
Renter	-1.72	0.47	-3.64	2.73 x 10-4
Median household income	0.07	0.11	0.62	0.54

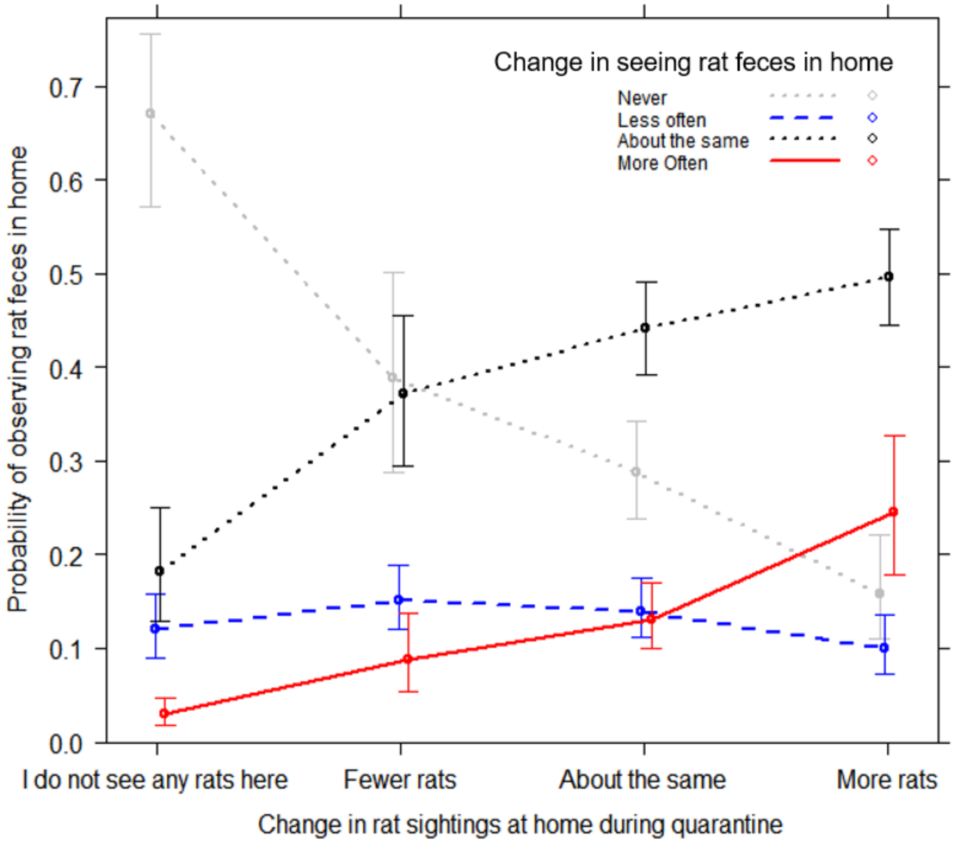

Figure S1: Relationship between survey respondents observing rats and rat feces in their home during quarantine.
